# Central auditory processing skill self-perception scale (CAPSSPS) and behavioral tests: a study in young adults with and without central auditory processing disorder

**DOI:** 10.1590/2317-1782/e20240212en

**Published:** 2025-10-20

**Authors:** Larissa Coradini, Hélinton Goulart Moreira, Michele Vargas Garcia

**Affiliations:** 1 Universidade Federal de Santa Maria – UFSM - Santa Maria (RS), Brasil.; 2 Programa de Pós-graduação em Distúrbios da Comunicação Humana, Departamento de Fonoaudiologia, Universidade Federal de Santa Maria – UFSM - Santa Maria (RS), Brasil.

**Keywords:** Surveys and Questionnaires, Auditory Processing, Young Adult, Hearing Tests, Hearing

## Abstract

**Purpose:**

To analyze the results of the Central Auditory Processing Disorder. Central Auditory Processing Skill Self-Perception Scale (CAPSSPS) and compare different auditory skills in young adults with and without Central Auditory Processing Disorder (CAPD).

**Methods:**

Cross-sectional and prospective study. Thirty-two individuals participated in the study, who were native Brazilian Portuguese speakers, non-bilingual, non-musicians, and not exposed to noise, with normal results in basic audiological assessments, no cognitive or otological complaints, with or without difficulties related to Central Auditory Processing, divided into two groups: participants without CAPD (G1) and participants with CAPD (G2). All participants underwent Anamnesis, Visual Inspection of the External Auditory Canal, Pure Tone Audiometry, Speech Audiometry, Acoustic Immittance Measures, Behavioral Tests of Central Auditory Processing, and the CAPSSPS questionnaire.

**Results:**

There was a significant difference between the groups with and without CAPD when comparing the CAPSSPS questionnaire and the Test of Frequency Pattern (TPF) - Auditec, Masking Level Difference (MLD), and the Gaps in Noise (GIN) tests for the left ear.

**Conclusions:**

The CAPSSPS questionnaire demonstrated the possibility of screening for other altered auditory skills, in addition to auditory closure and temporal resolution in young adult populations, including temporal ordering for frequency and binaural interaction. Furthermore, young adult individuals showed greater alterations in auditory skills of temporal resolution, temporal ordering for frequency, and poorer performance in binaural interaction skill.

## INTRODUCTION

Central auditory processing (CAP) refers to the effectiveness and efficiency with which the central auditory nervous system (CANS) uses sound information^(1)^. When there is a deficit in the processing of auditory signals throughout the SNAC, including one or more areas of auditory discrimination, binaural and temporal processing, it is called Central Auditory Processing Disorder (CAPD)^(2)^.

CAPD is a disorder related to speech perception deficits, in which alterations in one or more auditory skills can already characterize it^(2,3)^. Studies show the importance of evaluation in different age groups, given that individuals with CAPD may show behaviors related to difficulty understanding speech in noise, recurrent requests for repetition, as well as reduced attention and memory for verbal commands. In this sense, they may have speech disorders, language changes, impaired literacy, reduced academic performance and social behavior disorders, as well as difficulty discriminating, locating, recognizing, recording and/or understanding sound stimuli presented^(4)^.

In order to assertively measure CAP, national and international guidelines recommend associating and relating self-perceived hearing performance scales/questionnaires with behavioral and electrophysiological tests^(2-5)^. The prerogative is that through a complete evaluation, a diagnosis is made that reflects the individual's real conditions. Questionnaires and *checklists* are tools that can be used for this, since they provide relevant information on everyday situations related to the functioning of the auditory system, and can be obtained from the individual's own reports or from family members and/or teachers^(6)^.

A recent literature review^(6)^ aimed to identify CAP screening questionnaires available in Brazil for the Portuguese language, concluding that there is little national literature on the subject, highlighting the difficulty in identifying and/or the absence of screening instruments in questionnaire or checklist format aimed at adults and the elderly^(6)^.

Among the most widely used instruments are the Scale of Auditory Behaviors (SAB) and the *Children's Auditory Performance Scale* (CHAPS), aimed at children, and the Auditory Processing Domains Questionnaire (APDQ) for children and adolescents^(6)^. Of the national questionnaires, these are the only ones that cover all the auditory skills of CAP. In this context, the Central Auditory Processing Skills Self-Perception Scale (EAPAC) was recently created^(7).^demonstrating the possible performance related to just two auditory skills in the adult population: auditory closure and temporal resolution.

Given the impact of CAPD and the lack of instruments that cover all auditory skills in young adults, this study is justified. Also, due to the scarcity of validated instruments for the national population that contribute to the diagnosis of CAPD, allowing the measurement of self-perception in the population studied, in a clear, fast, assertive and effective way, helping in the speech therapy clinic. The hypothesis of the study is that the EAPAC can contribute to measuring auditory performance in relation to other auditory skills beyond those initially proposed in the questionnaire.

Therefore, the aim of this study was to analyze the results of the EAPAC self-perception questionnaire and compare the different hearing abilities in young adults with and without CAPD.

## METHOD

### Study design

This is a cross-sectional, prospective study, approved by the Research Ethics Committee (CEP) of the Federal University of Santa Maria (UFSM) under the number 56038322100005346. All study participants signed a Free and Informed Consent Form (FICF) to clarify the risks and benefits of their participation.

Inclusion criteria were: individuals of both sexes; aged between 18 and 35 years; educated (with higher education completed or in progress - equal to or greater than 13 years of schooling); mother tongue Brazilian Portuguese; hearing thresholds within normal range in all conventionally assessed frequencies - 250 to 8000Hz^(8)^; middle ear integrity and contralateral stapedial acoustic reflexes present at normal levels bilaterally; no cognitive complaints; with or without CAP-related difficulties.

We excluded individuals who tested positive for COVID-19 at any time (self-reported or proven by presenting the RT-PCR test), with chronic tinnitus perception; evident or diagnosed neurological or psychiatric impairment, a history of head trauma; complaints of dizziness; continuous exposure to noise or musical practice, as well as bilingual individuals.

### Participants

Participants were recruited by publicizing the research on the social networks of the school clinic and the researchers, from April 2023 to January 2024. Forty-six participants were seen, one (2.17%) was excluded for not being a Brazilian Portuguese speaker, nine (19.56%) for having altered perception of hearing abilities (with normal behavioral tests), one (2.17%) for having tested positive for COVID-19 and three (6.52%) for a diagnosis of hearing loss in isolated frequencies, not obtaining a quatritonal mean (QM) grade. All the participants were advised of the findings of the tests and, if they were interested in undergoing rehabilitation, were referred for treatment at the same institution.

The final sample consisted of 32 participants who met the inclusion criteria and were divided into two groups:

- Group 1 (G1) made up of 15 individuals with no Central Auditory Processing Disorder (CAPD) and normal EAPAC scores (three males and 12 females), aged between 18 and 32 years (mean: 21.63 years) and with between 13 and 22 years of schooling (mean: 15.04), i.e. above the third level (with higher education completed or in progress);
- Group 2 (G2) made up of 17 individuals with Central Auditory Processing Disorder (CAPD) (six males and 11 females), aged between 18 and 30 years (mean: 23.64 years) and with schooling between 13 and 22 years (mean: 16.29), i.e. also above the third level.

The groups were paired in terms of age, gender and schooling and analyzed using the *Mann-Whitney* U-test, with no statistically significant differences between them, as shown in Table 1.

**Table 1:** Analysis of the variables gender, age and schooling between the two groups

VARIABLES	GROUP	N	AVERAGE	SD	P-VALUE
SEX	G1	15	12W - 3M	-	0.345
	G2	17	11W - 6M	-	
AGE	G1	15	21.63	-	**0.083**
	G2	17	23.64	-	
SCHOOLING	G1	15	15.4	-	0.167
	G2	17	16.29	-	

**Caption:** G1= Group without CAPD; G2= Group with CAPD; W= women; M= men; N = number of individuals; SD = standard deviation

### Methodological design

The assessments were subdivided into sampling procedures and research procedures. The order in which they were carried out is described below, taking around one hour and 30 minutes.

### Procedures for sample composition

**Semi-structured anamnesis:** everyone answered the initial interview, collecting identification data, questions related to hearing, general health and eligibility criteria.
**Visual inspection of the external acoustic meatus:** this was carried out using a *Mikatos* TK otoscope, in order to ascertain the necessary conditions for the examination, as well as the possible need for referral to an otorhinolaryngologist.
**Pure tone audiometry (PTA):** this took place in an acoustically treated booth, using an R-27A- Resonance audiometer and TDH-39 headphones. Hearing thresholds were considered to be within normal limits when the thresholds for the frequencies conventionally assessed (250Hz to 8000Hz) were equal to or lower than 19 dBHL^(8)^.
**Logoaudiometry:** the same headphones and audiometer were used as for the ATL. The Speech Recognition Threshold (SRT) and then the Speech Recognition Percentage Index (SRPI) were investigated viva voce. Twenty-five monosyllable words were presented to the individual, and the result was considered normal when the percentage of correct answers was equal to or greater than 92%^(8)^.
**Acoustic immittance measurements:** two tests were carried out: the tympanometric curve, as classified by Jerger, Jerger and Mauldin (1972), and the stapedial acoustic reflexes contralaterally, at frequencies of 500 to 4000 Hz, as referenced by Jerger and Jerger (1989). The responses were obtained using an *Interacoustics* AT235 device and a TDH-39 headset^(8)^.

### Research procedures

#### Behavioral Tests of Central Auditory Processing

Behavioral Tests of Central Auditory Processing included the Digits Dichotic Test (DDT) - binaural integration stage, Pitch Pattern Sequence (PPS) - Auditec, Speech in Noise (SR) - signal-to-noise ratio +5dB ipsilateral, *Masking Level Difference* (MLD) and the *Gaps in Noise (GIN*) applied monaurally (track 1). These tests were selected in order to meet the minimum test battery suggested in accordance with national recommendations (CFFa^(2)^; ABA^(3)^).

All the tests were carried out at 40 dBSPL above the individual's tritonal mean (MTT), as the regulatory bodies suggest the possibility of 40 dBSPL above the tritonal mean, the same technique as the SRPI, since they do not reduce peripheral acuity^(9)^. All the behavioral tests were carried out in an acoustically treated booth, using the headphones and audiometer already mentioned, connected to a notebook.

National recommendations were taken into account (CFFa^(2)^; ABA^(3)^), where an altered test is considered a CAPD.

**Dichotic Digits Test (DDT):** used to assess the auditory ability of binaural integration. To analyze the results, the number of errors was added up and multiplied by 2.5%, then subtracted from 100 to find the percentage of correct answers for each ear. Responses equal to or greater than 95% were considered normal^(10)^.
**Pitch Pattern Sequence (PPS) - Auditec:** The PPS(adult version) was used to assess the auditory ability of temporal ordering for non-verbal sounds, with a binaural presentation, and a test strip with 30 stimuli. To analyze the results, the hits were added up and a simple rule of three was used to obtain the percentage, with 86.6% or more hits being used as the normal value^(11)^.
**Speech in Noise (SR):** used to assess auditory closure ability for verbal sounds. It was performed monaurally, with ipsilateral white noise, at an S/N ratio of 5 dBHL, i.e. the speech was 5 dBHL more intense than the noise. The normality standard used was 68% in the first ear presented (right ear) and 72% in the second ear presented (left ear)^(10)^.
***Masking Level Difference* (MLD):** used to assess hearing ability in binaural interaction. To analyze the results, the number of hits in the homophase and antiphase conditions must be counted and then the value converted into the table in the test protocol. The average will be the difference between the homophase and antiphase conditions. Normal values of 8 dB or more were used as a reference^(11)^.
***Gaps in Noise (GIN)*:** the GIN was used to assess auditory temporal resolution ability, monaurally. In order to analyze the final percentage of correct answers, the gap detection threshold was considered to be the smallest gap perceived by the patient in 50% of the times it was presented, and up to 5ms was considered to be normal^(12)^. Only band 1 was used, in both ears.

#### Central Auditory Processing Skills Self-Perception Scale (EAPAC)

The Central Auditory Processing Ability Self-Perception Scale was used as a self-assessment questionnaire^(7).^which consists of 13 questions and can be answered by individuals aged between 17 and 55. Thus, 12 questions have a "yes" answer, equivalent to one point, or a "no" answer, equivalent to 0 points. Question 13 also asks whether the individual went to private school (0 points) or public school (1 point).

The questions involve the perception of problems in detecting the acoustic stimulus, localization and lateralization of the sound source, recognition and discrimination of the acoustic stimulus, selective and sustained attention to the acoustic stimulus and short-term memory related to the acoustic stimulus. Also, to identify whether there are difficulties in perceiving sounds in time, difficulties in hearing and understanding speech in noisy situations and whether they have or have had academic difficulties related to concentration, memory, planning or learning at any point during their higher education course.

To analyze the EAPAC, the points obtained were added together, resulting in a total score. Scores lower than four were considered normal, scores equal to or higher than five are suggestive of altered auditory closure ability and scores equal to or higher than six are suggestive of altered temporal resolution ability^(7)^.

### Data analysis

In the statistical analysis, an investigation into the normality of the variables was carried out using the Kolmogorov-Smirnov test, which showed a non-normal distribution. The *Mann-Whitney* U-test was then used to compare the groups, with a significance value of p-value < 0.05.

## RESULTS

Table 2 shows a description and comparison of the EAPAC questionnaire and CAP behavioral tests between the groups without and with CAPD. There was a significant difference in the EAPAC questionnaire and in the PPS, MLD and GIN behavioral tests.

**Table 2:** Description and comparison of the EAPAC questionnaire and CAP behavioral tests between the groups

EAPAC/CAP tests	Group	N	Mean	SD	Minimum	Maximum	P-value
EAPAC	G1	15	2.06	1.22	0	4	**0.008** *
	G2	17	4.58	2.64	1	8	
SR RE	G1	15	88.53	9.66	72	100	0.688
	G2	17	86.70	11.11	64	100	
SR LE	G1	15	90.66	7.80	76	100	0.848
	G2	17	89.17	10.56	64	100	
DDT RE	G1	15	98.16	2.90	92.5	100	0.550
	G2	17	97.5	2.85	87.5	100	
DDT LE	G1	15	98.81	1.87	95	100	0.376
	G2	17	99.41	1.09	97.5	100	
GIN RE	G1	15	4.2	1.01	2	6	0.290
	G2	17	4.88	1.69	2	8	
GIN LE	G1	15	3.93	0.88	2	5	0.036*
	G2	17	4.82	1.18	3	8	
PPS	G1	15	95.94	3.13	90	100	0.009*
	G2	17	82.7	15.26	53.33	100	
MLD	G1	15	14.4	2.94	8	18	0.003*
	G2	17	9.68	5.33	2	20	

* = statistically significant difference

**Caption:** G1= Group without CAPD; G2= Group with CAPD; RE= right ear; LE= left ear; N = number of individuals; SD = standard deviation

**Figure 1:**
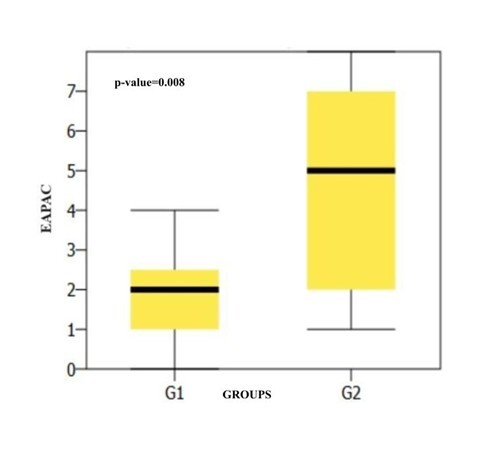
Comparison of the EAPAC questionnaire between the groups

Figure 1 shows the *box plot* comparing the results of the EAPAC questionnaire between the groups, with significant differences.

**Caption:** G1= Group without CAPD; G2= Group with CAPD

**Figure 2:**
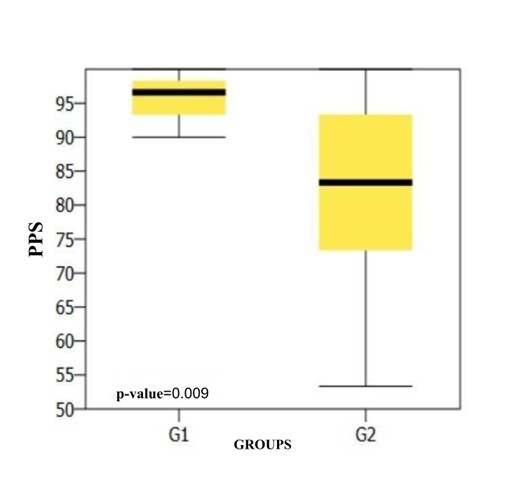
Comparison of the PPS results between the groups

Figure 2 shows a comparison of the results of the PPS behavioral test between the groups.

**Caption:** G1= Group without CAPD; G2= Group with CAPD

**Figure 3:**
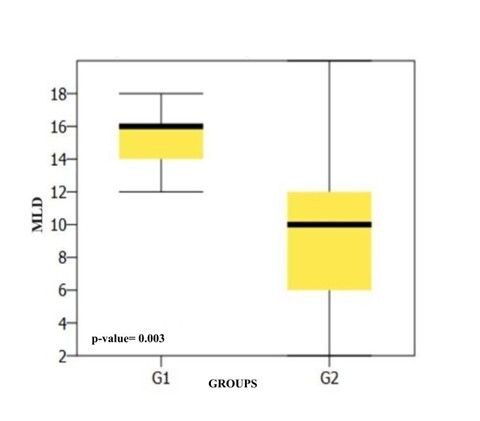
Comparison of MLD results between groups

Figure 3 shows the *box plot* comparing the results of the MLD behavioral test between the groups.

**Caption:** G1= Group without CAPD; G2= Group with CAPD

**Figure 4:**
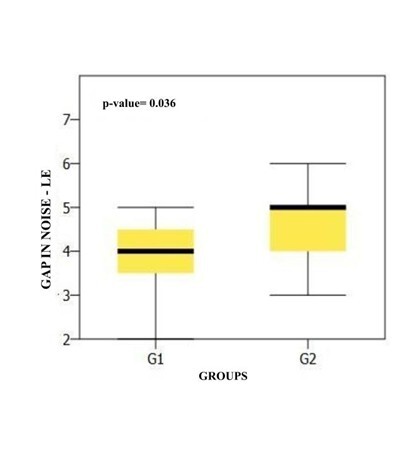
Comparison of left ear GIN results between groups

When comparing GIN performance by ear between the groups, Figure 4 shows that there was a statistically significant difference for the left ear.

**Caption:** G1= Group without CAPD; G2= Group with CAPD

## DISCUSSION

The use of self-assessment questionnaires has been widely cited in the literature as part of the diagnostic assessment battery for CAPD^(6-13)^. In this context, it is worth noting that the authors of the EAPAC themselves have suggested the importance of future research into the use and expansion of behavioral tests for the questionnaire^(7)^, which is in line with the aim of this study.

The findings of the present study corroborate the hypothesis that the EAPAC can contribute to assessing auditory performance in relation to other auditory skills besides auditory closure and temporal resolution, which the questionnaire was initially designed to address. These findings can be seen in Table 2, with significant differences between the groups and better scores in the skills assessed for G1 (without CAPD).

Worse scores, with significant differences, were observed in the EAPAC for individuals with CAPD. These findings corroborate other studies which have also shown altered performance on the scales applied to individuals with altered hearing abilities^(13,14)^. These findings are justified by the fact that the EAPAC is a questionnaire that covers aspects related to academic difficulties, regarding the school institution, possible executive deficits, attention, memory and performance in daily listening situations that are related to various auditory skills^(7)^. It should be noted that these issues may be impaired and self-perceived in individuals with altered hearing skills, given that an effective system is needed for adequate performance.

In the present study, significant differences were observed between the groups in binaural interaction skills and temporal aspects, measured by the PPS, GIN-LE and MLD tests (Figures 1, 2 and 3)^(15)^. The auditory skills measured by the PPS and MLD are not described as being possible to track by the EAPAC questionnaire, but the specialized literature shows these to be reliable and important tests for proper sound signal processing^(16,17)^. These skills are complex and play an essential role in the perception of continuous speech and its isolated parts, in learning and understanding language. They are therefore a prerequisite for language skills, as well as for the acquisition of reading and writing and for performance in challenging listening situations^(18)^. In this sense, these skills could be added to the existing protocol, due to their importance and the findings.

It is noteworthy that only differences were observed for the LE GIN in this study. This finding was justified in a study^(19)^, this difference may be related to perceptual asymmetry as a result of the stimulus used^(19)^.

The DDT has recently been described as a screening method for CAPD in basic audiological assessment, since its performance was associated with the SAB self-perception questionnaire in children aged 8 to 11 years^(20)^. These findings were not observed in the present study. Studies have shown that the DDT in adults can be used to detect any abnormality in brain function, attention, working memory and impairment of executive functions in patients^(21)^. Thus, its application focuses on measuring the advantage of the ears and possible binaural interference, neurological conditions such as unilateral stroke and psychiatric disorders such as schizophrenia^(21)^. The aforementioned findings support the results of the present study, since the population studied were young adults with a high level of education and no cognitive complaints. In view of the DDT data, it is suggested that these conditions be measured using the dichotic sentence test in order to obtain the real performance of adults^(22)^.

No statistically significant difference was found between the groups for auditory closure ability, as seen in the SN test, even though the same methodology was used as in the original article. The study by Sanguebuche et al.^(11)^ found that auditory closure ability performed less well when comparing groups aged 18-29 and 30-58. Therefore, since the age range in the EAPAC questionnaire was 18 to 51 years old^(7)^ and in the present study the age range was only 18 to 32 years old, this justifies the lack of significance between the groups.

Therefore, in view of the data presented here, observing the group without CAPD and the group with CAPD, with the aim of verifying how the EAPAC works in relation to other auditory skills than those already explained by the authors of the study, shows that the EAPAC questionnaire becomes even more relevant for young adults. In addition, in light of the findings of this study, the EAPAC can be used to screen for other altered auditory skills, in addition to auditory closure and temporal resolution, such as temporal ordering for frequency and binaural interaction.

In view of the scarcity of instruments in the literature that cover all auditory abilities in young adults, the results of this study contribute to clinical practice in the search for a clear, fast, assertive and effective measurement of self-perception in this population, as well as in defining the risk of CAPD.

### Limitations of the study

A limitation of this study was the impossibility of carrying out the cognitive screening due to the length of the audiological and auditory processing assessment, which was not feasible in two sessions.

## CONCLUSION

The EAPAC questionnaire demonstrated the possibility of screening for altered auditory skills other than auditory closure and temporal resolution in young adults, namely temporal ordering and binaural interaction.

In addition, young adults showed greater alteration in the auditory skills of temporal resolution, temporal ordering for frequency and worse performance in the skill of binaural interaction.
