# Supplementary material for: Central auditory processing skill self-perception scale (CAPSSPS) and behavioral tests: a study in young adults with and without central auditory processing disorder
Source: Codas. 2025 Oct 20;37(5):e20240212. doi: 10.1590/2317-1782/e20240212en (PMC12617280; doi:10.1590/2317-1782/e20240212en)
Supplement: Translation

# Translation: Escala de autopercepção de habilidades do processamento auditivo central (EAPAC) e testes comportamentais: um estudo em adultos jovens com e sem transtorno do processamento auditivo central

**DOI:** 10.1590/2317-1782/e20240212pt

**Authors:** Larissa Coradini, Hélinton Goulart Moreira, Michele Vargas Garcia

**Affiliations:** 1 Universidade Federal de Santa Maria – UFSM - Santa Maria (RS), Brasil.; 2 Programa de Pós-graduação em Distúrbios da Comunicação Humana, Departamento de Fonoaudiologia, Universidade Federal de Santa Maria – UFSM - Santa Maria (RS), Brasil.

**Keywords:** Inquéritos e Questionários, Processamento Auditivo, Adulto Jovem, Testes Auditivos, Audição

## Abstract

**Objetivo:**

Analisar o resultado da Escala de Autopercepção de Habilidades do Processamento Auditivo Central (EAPAC) e comparar as diferentes habilidades auditivas em adultos jovens com e sem Transtorno do Processamento Auditivo Central (TPAC).

**Método:**

Estudo de caráter transversal e prospectivo. Participaram do estudo 32 indivíduos, falantes do portugues brasileiro, não bilingues, musicistas ou expostos ao ruído, com normalidade na avaliação audiológica básica, sem queixas cognitivas e otológicas, com ou sem dificuldades relacionadas ao Processamento Auditivo Central (PAC), distribuídos em dois grupos: participantes sem TPAC (G1) e participantes com TPAC (G2). Todos foram submetidos a Anamnese, Inspeção Visual do Meato Acústico Externo, Audiometria Tonal Liminar, Logoaudiometria, Medidas de imitância acústica, Testes Comportamentais do Processamento Auditivo Central e ao questionário EAPAC.

**Resultados:**

Houve diferença significativa entre os grupos com e sem TPAC, quando comparado o questionário EAPAC e nos testes Teste Padrão de Frequência(TPF) - Auditec, Masking Level Difference(MLD) e o Gap in Noise (GIN) para a orelha esquerda.

**Conclusão:**

O questionário EAPAC demonstrou a possibilidade de rastrear outras habilidades auditivas alteradas, além de fechamento auditivo e resolução temporal no público adulto jovem, sendo elas ordenação temporal e interação binaural. Ademais, os indivíduos adultos jovens apresentaram maior alteração nas habilidades auditivas de resolução temporal, ordenação temporal e pior desempenho na habilidade de interação binaural.

## INTRODUÇÃO

O Processamento Auditivo Central (PAC) refere-se à eficácia e eficiência com que o sistema nervoso auditivo central (SNAC) utiliza a informação sonora^(1)^. Quando ocorre um déficit no processamento de sinais auditivos ao longo do SNAC, incluindo uma ou mais áreas de discriminação auditiva, processamento binaural e temporal, denomina-se Transtorno do Processamento Auditivo Central (TPAC)^(2)^.

O TPAC trata-se de um distúrbio relacionado aos déficits da percepção de fala, do qual a alteração em uma ou mais habilidades auditivas já podem caracterizá-lo^(2,3)^. Estudos demonstram a importância da avaliação nas diferentes faixas etárias, tendo em vista que indivíduos com TPAC podem apresentar comportamentos relacionados à dificuldade de compreensão de fala no ruído, solicitações recorrentes de repetição, bem como redução atencional e memória para comandos verbais. Nesse sentido, podem apresentar distúrbios de fala, alterações de linguagem, prejuízos na alfabetização, redução do desempenho acadêmico e distúrbios de comportamento social, além de dificuldade para discriminar, localizar, reconhecer, registrar e/ou compreender estímulos sonoros apresentados^(4)^.

Para que a mensuração assertiva do PAC ocorra, diretrizes nacionais e internacionais recomendam que sejam associados e relacionados escalas/questionários de autopercepção do desempenho auditivo com testes comportamentais e eletrofisiológicos^(2-5)^. A prerrogativa é que por meio de uma avaliação completa ocorra um diagnóstico que reflete as reais condições do indivíduo. Questionários e *checklists* são instrumentos que podem ser utilizados para tal, uma vez que trazem informações relevantes sobre situações do cotidiano relacionadas a esse funcionamento do sistema auditivo, podendo ser obtidas de relatos do próprio indivíduo ou por familiares e/ou professores^(6)^.

Uma revisão de literatura recente^(6)^ objetivou identificar questionários para triagem do PAC disponíveis no Brasil para a língua portuguesa, concluindo que há uma escassa literatura nacional relacionadas à temática, destacando a dificuldade de identificação e/ou ausência de instrumentos de triagem em formato de questionário ou checklist voltados para adultos e idosos^(6)^.

Dentre os instrumentos mais utilizados encontra-se o uso da Escala de Comportamentos Auditivos (SAB) e o *Children's Auditory Performance Scal*e (CHAPS), voltados ao público infantil, e o Questionário de Domínios do Processamento Auditivo (APDQ) para crianças e adolescentes^(6)^. Dos questionários nacionais, estes são os únicos que abrangem todas as habilidades auditivas do PAC. Nesse contexto, recentemente foi criada a Escala de Autopercepção de Habilidades do Processamento Auditivo Central (EAPAC)^(7)^, demonstrando o possível desempenho relacionado apenas para duas habilidades auditivas na população adulta: fechamento auditivo e resolução temporal.

Tendo em vista os impactos advindos do TPAC e a carência de instrumentos que contemplem todas as habilidades auditivas em adultos jovens, justifica-se a presente pesquisa. Ainda, devido à escassez de instrumentos validados para população nacional e que contribuem no diagnóstico do TPAC, permitindo a mensuração da autopercepção na população estudada, de forma clara, rápida, assertiva e eficaz, auxiliando na clínica fonoaudiológica. A hipótese do estudo centra-se em que a EAPAC possa contribuir para a mensuração do desempenho auditivo relacionada a outras habilidades auditivas além das que o questionário se propõe inicialmente.

Portanto, o objetivo deste estudo foi analisar o resultado do questionário de autopercepção EAPAC e comparar as diferentes habilidades auditivas em adultos jovens com e sem TPAC.

## MÉTODO

### Desenho do estudo

Trata-se de um estudo de caráter transversal e prospectivo, aprovado pelo Comitê de Ética em Pesquisa (CEP), da Universidade Federal de Santa Maria (UFSM), sob o número 56038322100005346. Todos os participantes do estudo assinaram o Termo de Consentimento Livre e Esclarecido (TCLE), pretendendo esclarecer sobre os riscos e benefícios da sua participação.

Foram estabelecidos como critérios de inclusão: indivíduos de ambos os sexos; idades entre 18 e 35 anos; escolarizados (com ensino superior completo ou cursando - igual ou superior há 13 anos de escolaridade); com língua materna o português brasileiro; limiares auditivos dentro dos padrões de normalidade em todas as frequências convencionalmente avaliadas - 250 a 8000Hz^(8)^; integridade de orelha média e reflexos acústicos estapedianos contralaterais presentes em níveis normais bilateralmente; sem queixas cognitivas; com ou sem dificuldades relacionadas ao PAC.

Foram excluídos indivíduos que positivaram para COVID-19 em qualquer momento (autorrelatado ou comprovados por meio da apresentação do teste RT-PCR), com percepção de zumbido crônico; comprometimento neurológico ou psiquiátrico evidentes ou diagnosticados, histórico de trauma cranioencefálico; queixas de tontura; exposição contínua ao ruído ou a prática musical, bem como indivíduos bilíngues.

### Participantes

Os participantes foram captados por meio da divulgação da pesquisa nas redes sociais da clínica escola e dos pesquisadores, no período de abril de 2023 a janeiro de 2024. Foram atendidos 46 participantes, um (2,17%) foi excluído por não ser falante do português brasileiro, nove (19,56%) por terem percepção das habilidades auditivas alteradas (com testes comportamentais normais), um (2,17%) por ter positivado para COVID-19 e três (6,52%) por diagnóstico de perda auditiva em frequências isoladas, não obtendo grau pela média quatridonal (MQT). Todos os participantes foram orientados quanto aos achados nos exames, e caso tivessem interesse em realizar reabilitação, encaminhados para tratamento na mesma instituição de avaliação.

Assim, a casuística final foi composta por 32 participantes que atenderam aos critérios de inclusão, os quais foram distribuídos em dois grupos:

- Grupo 1 (G1) composto por 15 indivíduos sem Transtorno do Processamento Auditivo Central (TPAC) e com normalidade no EAPAC (três do sexo masculino e 12 do sexo feminino), com idades entre 18 e 32 anos (média: 21,63 anos) e com escolaridade entre 13 e 22 anos (média: 15,04), ou seja, acima do terceiro nível (com ensino superior completo ou cursando);
- Grupo 2 (G2) composto por 17 indivíduos com Transtorno do Processamento Auditivo Central (TPAC) (seis do sexo masculino e 11 do sexo feminino), com idades entre 18 e 30 anos (média: 23,64 anos) e com escolaridade entre 13 e 22 anos (média: 16,29), ou seja, também acima do terceiro nível.

Os grupos foram pareados quanto a idade, sexo e escolaridade e analisados pelo teste U de *Mann-Whitney*, sem diferenças estatisticamente entre eles, como demonstrado na Tabela 1.

**Tabela 1:** Análise das variaveis sexo, idade e escolaridade entre ambos os grupos

VARIÁVEIS	GRUPO	N	MÉDIA	DP	P-VALOR
SEXO	G1	15	12M - 3H	-	0,345
	G2	17	11M - 6H	-	
IDADE	G1	15	21,63	-	**0,083**
	G2	17	23,64	-	
ESCOLARIDADE	G1	15	15,4	-	0,167
	G2	17	16,29	-	

**Legenda:** G1= Grupo sem TPAC; G2= Grupo com TPAC; M= mulheres; H= Homens; N= número de indivíduos; DP= desvio padrão

### Delineamento metodológico

As avaliações foram subdivididas em procedimentos para composição amostral e procedimentos de pesquisa. A ordem de aplicação dos mesmos está descrita abaixo, contabilizando em torno de uma hora e 30 minutos.

### Procedimentos para composição amostral

**Anamnese semiestruturada:** todos responderam a entrevista inicial, sendo coletados dados de identificação, questões relacionadas à audição, bem como de saúde no geral e critérios de elegibilidade.
**Inspeção Visual do Meato Acústico Externo:** foi realizada com o otoscópio, modelo TK da marca *Mikatos*, a fim de averiguar as condições necessárias de realização do exame, assim como a possível necessidade de encaminhamento ao profissional otorrinolaringologista.
**Audiometria Tonal Liminar (ATL):** ocorreu em cabine acusticamente tratada, utilizando o audiômetro R-27A- Resonance e fones de ouvido TDH-39. Foram considerados limiares auditivos dentro dos padrões de normalidade quando os limiares das frequências convencionalmente avaliadas (250Hz a 8000Hz), estivessem igual ou inferior a 19 dBNA^(8)^.
**Logoaudiometria:** utilizou-se os mesmos fones de ouvido e audiômetro da ATL. Foram pesquisados à viva-voz, o Limiar de Reconhecimento de Fala (LRF) e posteriormente, o Índice Percentual de Reconhecimento de Fala (IPRF). Foram apresentadas 25 palavras monossílabas ao indivíduo, considerando como normal o resultado quando obtida uma porcentagem de acertos igual ou superior a 92%^(8)^.
**Medidas de Imitância Acústica:** realizou-se dois testes, curva timpanométrica, classificando por Jerger, Jerger e Mauldin (1972), e os reflexos acústicos estapedianos de modo contralateral, nas frequências de 500 a 4000 Hz, referenciando por Jerger e Jerger (1989). As respostas foram obtidas no equipamento AT235, da marca *Interacoustics* e fone tipo TDH-39^(8)^.

### Procedimentos de pesquisas

#### Testes Comportamentais do Processamento Auditivo Central

Foram realizados como Testes Comportamentais do Processamento Auditivo Central, o Teste Dicótico de Dígitos (TDD) - etapa de integração binaural, Teste Padrão de Frequência (TPF) - Auditec, Fala com Ruído (FR) - relação sinal-ruído +5dB ipsilateral, *Masking Level Difference* (MLD) e o *Gaps in Noise (GIN)* aplicado de forma monoaural (faixa 1). Estes testes foram selecionados, em busca de atender à bateria de testes mínimos sugeridos, de acordo com as recomendações nacionais (CFFa^(2)^; ABA^(3)^).

Todos os testes foram realizados com apresentação em 40 dBNS acima da média tritonal (MTT) do indivíduo, pois os órgãos regulamentadores trazem a possibilidade de 40 dBNS acima da média tritonal, mesma técnica do IPRF, uma vez que não apresentam redução da acuidade periférica^(9)^. Todos os testes comportamentais foram realizados em uma cabine tratada acusticamente, utilizando os fones de ouvido e audiômetro já citados, conectado a um notebook.

Levou-se em consideração as recomendações nacionais (CFFa^(2)^; ABA^(3)^), onde um teste alterado é considerado como TPAC.

**Teste Dicótico de Dígitos (TDD):** utilizado para avaliar a habilidade auditiva de integração binaural. Para a análise dos resultados, somou-se o número de erros e multiplicou-se por 2,5%, logo subtraiu-se de 100, encontrando-se a percentagem de acertos, para cada orelha. Considerou-se normalidade respostas iguais ou superiores a 95%.^(10)^.
**Teste Padrão de Frequência (TPF) - Auditec:** O TPF (versão adulto) foi utilizado para avaliar a habilidade auditiva de ordenação temporal para sons não verbais, com apresentação de forma binaural, sendo uma faixa teste com os 30 estímulos. Para a análise dos resultados, somou-se os acertos e fez-se uma regra de três simples para obter o percentual, sendo utilizado como valor de normalidade 86,6% ou mais de acertos ^(11)^.
**Fala com Ruído (FR):** utilizado para avaliar a habilidade auditiva de fechamento auditivo para sons verbais. Foi realizado de forma monoaural, com ruído branco de modo ipsilateral, em uma relação S/R de 5 dBNA, ou seja, a fala estava 5 dBNA mais intensa do que o ruído. O padrão de normalidade utilizado foi de 68% na primeira orelha apresentada (orelha direita) e 72% na segunda orelha apresentada (orelha esquerda)^(10)^.
***Masking Level Difference* (MLD):** utilizado para avaliar a habilidade auditiva de interação binaural, de forma binaural. Para a análise dos resultados, deve-se contar os acertos das condições homofásica, antifásica e após converter o valor na tabela que consta no protocolo do teste. A média será a diferença entre a condição homofásica e antifásica. Foi utilizada como referência os valores de normalidade de 8 dB ou mais^(11)^.
***Gaps in Noise (GIN)*:** o GIN foi utilizado para avaliar a habilidade auditiva de resolução temporal, de forma monoaural. Para analisar a porcentagem final de acertos, considerou-se o limiar de detecção de gap o menor gap percebido pelo paciente em 50% das vezes em que foi apresentado, sendo considerado padrão de normalidade até 5ms^(12)^. Foi utilizado somente a faixa 1, em ambas orelhas.

#### Escala de Autopercepção de Habilidades do Processamento Auditivo Central (EAPAC)

Como questionário de autoavaliação das habilidades auditivas foi aplicada a Escala de Autopercepção de Habilidades do Processamento Auditivo Central (EAPAC)^(7)^, a qual é composta por 13 questões e pode ser respondida por indivíduos com idades entre 17 e 55 anos. Dessa maneira, 12 questões são com alternativa de resposta “sim”, equivalente a um ponto, ou "não” equivalente a 0 pontos. Ainda, a pergunta 13 questiona se o indivíduo estudou em escola particular (0 pontos) ou pública (1 ponto).

As questões envolvem a percepção de problemas na detecção do estímulo acústico, localização e lateralização da fonte sonora, reconhecimento e discriminação do estímulo acústico, na atenção seletiva e sustentada do estímulo acústico e memória de curta duração relacionados ao estímulo acústico. Ainda, identificar se existem dificuldades para perceber os sons no tempo, dificuldades para ouvir e entender a fala em situações ruidosas e se apresenta ou já apresentou dificuldades acadêmicas relacionadas à concentração, memória, planejamento ou aprendizagem em algum momento do curso superior.

Para a análise da EAPAC, foram somados os pontos obtidos, resultando em uma pontuação total. Foi considerada normalidade escores menores que quatro, pontuações iguais ou superiores que cinco são sugestivos de alteração na habilidade de fechamento auditivo e iguais ou superiores que seis são sugestivos de alteração na habilidade de resolução temporal^(7)^.

### Análise dos dados

Na análise estatística, foi realizada uma investigação quanto à normalidade das variáveis, por meio do Kolmogorov-Smirnov, demonstrando uma distribuição não normal. Em seguida, foi utilizado o Teste utilizado o U de *Mann-Whitney* para a comparação entre os grupos, adotando como valor de significância p-valor < 0,05.

## RESULTADOS

Na Tabela 2, é apresentada uma descrição e comparação do questionário EAPAC e dos testes comportamentais do PAC entre os grupos sem e com TPAC. Observa-se diferença significativa no questionário EAPAC e nos testes comportamentais TPF, MLD e GIN.

**Tabela 2:** Descrição e comparação do questionário EAPAC e dos testes comportamentais do PAC entre os grupos

EAPAC/Testes PAC	Grupos	N	Média	DP	Mínimo	Máximo	P-valor
EAPAC	G1	15	2,06	1,22	0	4	0,008*
	G2	17	4,58	2,64	1	8	
FR OD	G1	15	88,53	9,66	72	100	0,688
	G2	17	86,70	11,11	64	100	
FR OE	G1	15	90,66	7,80	76	100	0,848
	G2	17	89,17	10,56	64	100	
TDD OD	G1	15	98,16	2,90	92,5	100	0,550
	G2	17	97,5	2,85	87,5	100	
TDD OE	G1	15	98,81	1,87	95	100	0,376
	G2	17	99,41	1,09	97,5	100	
GIN OD	G1	15	4,2	1,01	2	6	0,290
	G2	17	4,88	1,69	2	8	
GIN OE	G1	15	3,93	0,88	2	5	0,036*
	G2	17	4,82	1,18	3	8	
TPF	G1	15	95,94	3,13	90	100	0,009*
	G2	17	82,7	15,26	53,33	100	
MLD	G1	15	14,4	2,94	8	18	0,003*
	G2	17	9,68	5,33	2	20	

* = diferença estatisticamente significante

**Legenda:** G1= Grupo sem TPAC; G2= Grupo com TPAC; OD= orelha direita; OE= orelha esquerda; N= número de indivíduos; DP= desvio padrão

A Figura 1 apresenta o *box plot* da comparação dos resultados do questionário EAPAC entre os grupos, com diferenças significativas.

**Figura 1:** Comparação do questionário EAPAC entre os grupos

**Legenda:** G1= Grupo sem TPAC; G2= Grupo com TPAC

A comparação dos resultados do teste comportamental TPF entre os grupos, está apresentada na Figura 2.

**Figura 2:** Comparação do resultado do TPF entre os grupos

**Legenda:** G1= Grupo sem TPAC; G2= Grupo com TPAC

A Figura 3 apresenta o *box plot* da comparação dos resultados do teste comportamental MLD entre os grupos.

**Figura 3:** Comparação do resultado do MLD entre os grupos

**Legenda:** G1= Grupo sem TPAC; G2= Grupo com TPAC

Ao comparar o desempenho no GIN, por orelha, entre os grupos, pôde-se observar na Figura 4 que obteve-se diferença estatística significativa para a orelha esquerda.

**Figura 4:** Comparação do resultado do GIN da orelha esquerda entre os grupos

**Legenda:** G1= Grupo sem TPAC; G2= Grupo com TPAC

## DISCUSSÃO

O uso de questionários de autoavaliação tem sido amplamente citados na literatura como parte da bateria de avaliação diagnóstica para TPAC^(6-13)^. Nesse contexto, cabe salientar que os próprios autores da EAPAC sugeriram e colocaram a importância de futuras pesquisas relacionadas à utilização e ampliação de testes comportamentais para o questionário^(7)^, indo ao encontro do objetivo deste estudo.

Os achados do presente estudo corroboram com a hipótese de que a EAPAC possa contribuir para avaliar o desempenho auditivo em relação a outras habilidades auditivas para além das de fechamento auditivo e resolução temporal, das quais o questionário se propõe inicialmente. Tais constatações são observadas na tabela 2, com diferenças significativas entre os grupos e melhores escores nas habilidades avaliadas para G1 (sem TPAC).

Foram observadas piores pontuações, com diferenças significantes, na EAPAC para os indivíduos com TPAC. Esses achados corroboram com outros estudos que também demonstraram desempenhos alterados nas escalas aplicadas em indivíduos com alteração nas habilidades auditivas^(13,14)^. Tais constatações justificam-se devido a EAPAC ser um questionário que abrange aspectos relacionados a dificuldades acadêmicas, referentes à instituição escolar, aos possíveis déficits executivos, de atenção, memória e ao desempenho em situações de escuta diárias que estão relacionadas a diversas habilidades auditivas^(7)^. Destaca-se que estas questões podem estar prejudicadas e autopercebidas em indivíduos com alteração nas habilidades auditivas, tendo em vista que para um desempenho adequado, necessita-se de um sistema efetivo.

No presente estudo foram observadas diferenças significativas entre os grupos nas habilidades de interação binaural e nos aspectos temporais, mensuradas pelos testes TPF, GIN-OE e MLD (Figuras 1, 2 e 3)^(15)^. As habilidades auditivas mensuradas pelo TPF e MLD não estão descritas como possíveis de serem rastreadas pelo questionário EAPAC, porém a literatura especializada demonstra esses como testes que apresentam uma confiabilidade e de importância para o adequado processamento do sinal sonoro^(16,17)^. Tais habilidades são complexas e exercem funções essenciais na percepção da fala contínua e de suas partes isoladas, no aprendizado e na compreensão da linguagem. Assim, se constituem como um pré-requisito para habilidades linguísticas, bem como na aquisição da leitura e escrita e no desempenho nas situações de escuta desafiadoras^(18)^. Nesse sentido, tais habilidades podem ser acrescentadas ao protocolo já existente, devido a sua importância e pelos achados encontrados.

Destaca-se que foram observadas apenas diferenças para o GIN da OE na presente pesquisa. Esse achado foi justificado em um estudo^(19)^, do qual relatou a vantagem da orelha direita sobre a esquerda, do qual esse desempenho pode ser observado, ou seja, essa diferença pode estar relacionada à assimetria perceptual, em decorrência do estímulo empregado^(19)^.

O TDD recentemente foi descrito como um método de triagem do TPAC na avaliação audiológica básica, uma vez que houve associação no seu desempenho com o questionário de autopercepção SAB, em crianças de 8 a 11 anos^(20)^. Na presente pesquisa não foram observados esses achados. Estudos demonstram que o TDD, em adultos, pode ser usado ​​para detectar qualquer anormalidade no funcionamento cerebral, atenção, memória de trabalho e comprometimento das funções executivas nos pacientes^(21)^. Desse modo, a sua aplicação centra-se na mensuração da vantagem das orelhas e possíveis interferências binaurais, das condições neurológicas como acidente vascular cerebral unilateral e distúrbios psiquiátricos como esquizofrenia^(21)^. Os achados supracitados fundamentam os resultados do presente estudo, já que a população estudada foi de adultos jovens, de alta escolaridade e sem queixas cognitivas. Frente aos dados do TDD, sugere-se mensurar tais condições com o uso do teste dicótico de sentenças para que se obtenha o real desempenho nos adultos^(22)^.

Para a habilidade de fechamento auditivo, vista no teste FR, não foi encontrada diferença estatisticamente significativa entre os grupos, mesmo tendo sido utilizada a mesma metodologia do artigo original. O estudo de Sanguebuche et al.^(11)^ constatou que a habilidade de fechamento auditivo tem um desempenho menor quando compara-se grupos de 18 a 29 anos e de 30 a 58 anos. Dessa forma, como no questionário EAPAC a idade variou de 18 a 51 anos^(7)^ e no presente estudo as idades variaram apenas de 18 a 32 anos, justifica-se a não significância entre os grupos.

Portanto, diante dos dados aqui expostos, ao observar o grupo sem TPAC e o grupo com TPAC, objetivando verificar o funcionamento do EAPAC frente a outras habilidades auditivas que não as já expostas pelas autoras do estudo, demonstra que o questionário EAPAC torna-se ainda mais relevante para o público adulto jovem. Além disso, frente aos achados desse estudo, acrescenta-se a EAPAC a possibilidade de rastrear outras habilidades auditivas alteradas, para além de fechamento auditivo e resolução temporal, sendo elas ordenação temporal para frequência e interação binaural.

Tendo em vista, a escassez de instrumentos na literatura, que contemple todas as habilidades auditivas em adultos jovens, o resultado do presente estudo contribui para a prática clínica, em busca de uma mensuração da autopercepção na população de forma clara, rápida, assertiva e eficaz, bem como na definição do risco de TPAC.

### Limitações do estudo

Configura uma limitação deste estudo, a impossibilidade de realizar o rastreio cognitivo, devido a extensão do tempo de avaliação audiológica e do processamento auditivo, não sendo viável realizar em duas sessões.

## CONCLUSÃO

O questionário EAPAC demonstrou a possibilidade de rastrear outras habilidades auditivas alteradas, além de fechamento auditivo e resolução temporal no público adulto jovem, sendo elas ordenação temporal e interação binaural.

Ademais, os indivíduos adultos jovens apresentaram maior alteração nas habilidades auditivas de resolução temporal, ordenação temporal para frequência e pior desempenho na habilidade de interação binaural.
